# The *C. elegans* endoplasmic reticulum molecular chaperone ENPL-1 does not promote GLP-1/Notch signaling

**DOI:** 10.17912/micropub.biology.002171

**Published:** 2026-07-08

**Authors:** James L. Lissemore, Amanda Pancake, Shurong Fang, Eleanor M. Maine

**Affiliations:** 1 Department of Biology, John Carroll University, University Heights, OH, USA; 2 Department of Mathematics, Computer Science, and Data Science, John Carroll University, University Heights, OH, USA; 3 Department of Biology, Syracuse University, Syracuse, NY, USA

## Abstract

The *C. elegans* Notch receptor, GLP-1 , is essential for germline proliferation and early embryonic development. While the cytosolic chaperone HSP-90 promotes GLP-1 signaling in the germline, it is unknown whether the endoplasmic reticulum-resident Hsp90, ENPL-1 (GRP94/endoplasmin), also promotes GLP-1 signaling. We used RNAi knockdown of *enpl-1* in a sensitized *glp-1 ( bn18 )* background to investigate potential genetic interactions. RNAi-mediated knockdown of *enpl-1* did not enhance reduced GLP-1 /Notch signaling, and it caused a similar degree of early larval arrest in both wildtype and *glp-1 ( bn18 )* backgrounds. Thus, we did not detect a role for ENPL-1 in promoting GLP-1 /Notch signaling.

**Figure 1. enpl-1 knockdown causes larval arrest and does not enhance glp-1(bn18) germline or embryonic phenotypes:** **A)** Brood size, **B)** embryonic lethality, and **C)** larval arrest were determined for N2 wildtype and *glp-1 ( bn18 )* hermaphrodites treated with *gfp(RNAi)* (negative control) and *enpl-1 (RNAi)* . Horizontal lines within box plots indicate median; upper and lower ends of boxes indicate upper and lower quartiles; whiskers extend to minimum and maximum values. *gfp(RNAi)* N2 (GFP N2 ), n=9; *gfp(RNAi)* *glp-1 ( bn18 )* (GFP *bn18* ), n=11; *enpl-1 (RNAi)* N2 ( *enpl-1* N2 ), n=16; *enpl-1 (RNAi)* *glp-1 ( bn18 )* ( *enpl-1 bn18* ), n=10. * p<0.01; ** p<0.001; *** p<0.0001. **D-G)** Dissecting microscope images of 20X (left) and 60X (right) magnification of **D)** GFP N2 , **E)** GFP *bn18* , **F)** *enpl-1* N2 , **G)** *enpl-1 bn18* plates ~48 hours after removal of F1 parental hermaphrodite. For **F** ) and **G** ) 60X images, note that substantial numbers of arrested larvae had wandered off the lawn.

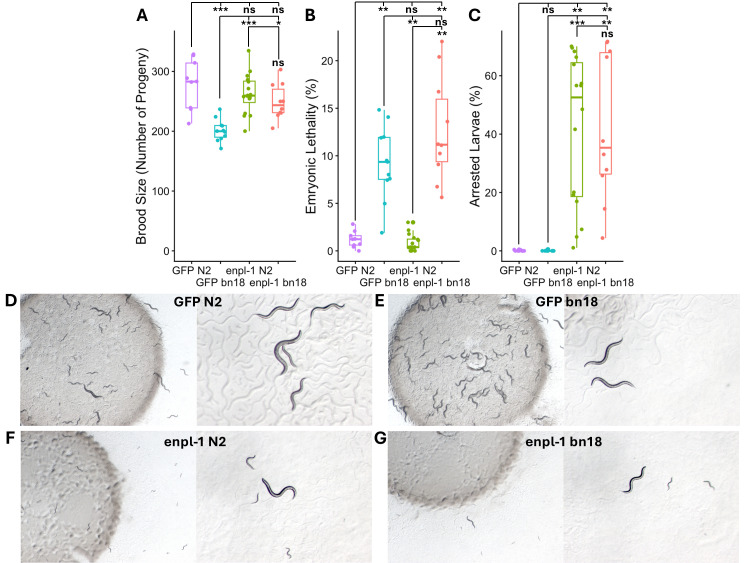



## Description

The *C. elegans* GLP-1 /Notch transmembrane receptor plays key roles in germline stem cell proliferation and early embryonic development (Maduro 2010; Hubbard and Schedl, 2019). We have previously shown that the molecular chaperone HSP-90 promotes germline GLP-1 /Notch signaling. Specifically, reduction of HSP-90 function enhances the germline stem cell proliferation defect observed in *glp-1 ( bn18 )* , a temperature-sensitive allele of *glp-1* (Qiao et al., 1995; Lissemore et al., 2018). These observations, along with evidence for a direct physical interaction between Hsp90 and Notch in mammalian cells (Deskin et al., 2016; Wang et al., 2017), are consistent with GLP-1 /Notch being a client protein of HSP-90 in *C. elegans* . In addition to cytosolic HSP-90 , *C. elegans* possesses an endoplasmic reticulum-specific molecular chaperone, ENPL-1 (GRP94/endoplasmin) (Podraza-Farhanieh et al., 2020). As a transmembrane receptor, GLP-1 /Notch is trafficked through the rough endoplasmic reticulum/Golgi endomembrane system (Zhou et al., 2022), leading us to ask whether the ENPL-1 chaperone, like HSP-90 , plays a role in GLP-1 /Notch signaling.

*glp-1 ( bn18 )* mutants have a strong germline proliferation defect at 25 °C, a mild germline proliferation defect at 20 °C, and essentially normal germline proliferation at 15 °C (Kodoyianni et al., 1992; Qiao et al., 1995). At 20 °C, *glp-1 ( bn18 )* provides a sensitized genetic background to identify other factors that either limit or promote GLP-1 /Notch signaling. Here we examined the effect of *enpl-1* knockdown in N2 and *glp-1 ( bn18 )* strains at 20 °C by measuring brood size and embryonic lethality to assess a possible role for *enpl-1* in GLP-1 /Notch signaling in the germline and during early embryonic development, respectively. *enpl-1 (RNAi)* was previously reported to cause larval arrest in the RNAi hypersensitive mutant *rrf-3 ( pk1426 )* (Billing et al., 2012). Therefore, we also examined whether larval arrest can occur in strains that have normal sensitivity to RNAi, i.e. N2 and *glp-1 ( bn18 )* .

We measured brood sizes of individual F1 N2 and *glp-1 ( bn18 )* hermaphrodites on *gfp(RNAi)* and *enpl-1 (RNAi)* feeding plates as well as embryonic lethality and larval arrest among F2 offspring. With respect to brood size, *enpl-1 (RNAi)* did not reduce brood size in either N2 or *glp-1 ( bn18 )* mutants compared to *gfp(RNAi)* negative controls ( Fig. 1A ). Therefore, *enpl-1* knockdown did not enhance the germline defect associated with *glp-1 ( bn18 )* raised at 20 °C. We do note a small but statistically significant increase in brood size in *enpl-1 (RNAi)* *glp-1 ( bn18 )* compared to *gfp(RNAi) glp-1 ( bn18 )* ( Fig. 1A ). Moreover, the brood size of *enpl-1 (RNAi)* *glp-1 ( bn18 )* was not statistically different from *enpl-1 (RNAi)* N2 .&nbsp; Because GLP-1 /Notch signaling is also required for embryonic development (Maduro 2010), we evaluated embryonic viability in *enpl-1 (RNAi* ). Embryonic lethality was significantly higher in *glp-1 ( bn18 )* mutants than in N2 for both *gfp(RNAi)* and *enpl-1 (RNAi)* , and the percent embryonic lethality was not statistically different in the two genetic backgrounds ( Fig. 1B ). That is, no interaction between *glp-1 ( bn18 )* and *enpl-1 (RNAi)* was seen with respect to embryonic lethality. &nbsp;Finally, *enpl-1 (RNAi)* caused early larval arrest at approximately the L2 stage in both N2 and *glp-1 ( bn18 )* based on larval size ( Fig. 1C- G). This *enpl-1 (RNAi)* larval arrest was not enhanced in *glp-1 ( bn18 )* mutants compared to N2 ( Fig. 1C ). These results confirm that *enpl-1* function was reduced in *enpl-1 (RNAi)* and that the negative results seen for brood size and embryonic lethality were not due to ineffective knockdown. Furthermore, our data show that an RNAi hypersensitive strain is not needed to observe the *enpl-1* larval arrest phenotype.

Taken together, our data do not reveal a substantial genetic interaction between *enpl-1* and *glp-1 .* We conclude that the ENPL-1 endoplasmic reticulum molecular chaperone plays little or no role in GLP-1 /Notch signaling in either germline or embryonic development in *C. elegans .*

## Methods

**Nematode strains and worm maintenance.** Worms were maintained using standard methods as previously described (Lissemore et al., 2018).

**RNAi feeding experiments.** RNAi was performed with modification of the feeding method (Timmons et al., 2001). Bacterial strains carrying plasmids expressing *gfp* dsRNA or *enpl-1* dsRNA (for *gfp(RNAi)* and *enpl-1 (RNAi)* , respectively) were inoculated into LB-ampicillin for overnight growth at 37 °C with shaking. Twenty μl of bacterial culture was spotted onto 35 mm plates of NGM-Lite medium containing ampicillin (100 μg/ml) and IPTG (1 mM). Bacterial lawns were allowed to grow for at least 1 day at 15 °C before use. Seeded plates were stored at 15 °C and used within 10-11 days.

Feeding experiments used the following schedule (plates were incubated at 20 °C):

Day 1: Single L4 hermaphrodites (P0) were transferred to RNAi feeding plates

Day 2: Worms were replica plated to fresh RNAi feeding plates

Day 3: Parental worms were removed from plates

Day 5: L4 offspring (F1) were cloned out on fresh RNAi feeding plates to determine A) brood size, B) percent embryonic viability, and C) percent arrested larvae.

A) Brood sizes of F1 hermaphrodites were determined by manually counting the number of embryos and L1 worms present ~24 hours after the F1 parent was initially placed on a plate, which was shortly after the parent had been transferred to a fresh plate. F1 parents were transferred until they no longer produced embryos (typically 4-5 days).

B) Embryonic viability of F2 progeny was determined by manually counting the number of unhatched embryos on plates ~24 hours after transfer of the parent worm.

C) Larval arrest of F2 progeny was determined by manually counting the number of arrested larvae (which appeared to be the size of L1/L2 worms) on plates ~48 hours after transfer of the parent worm.

**Statistical Analysis**

A traditional One-way ANOVA with the Tukey HSD Post-Hoc Test was used to determine the differences in brood size among four groups: *gfp(RNAi)* N2 , *gfp(RNAi)* *glp-1 ( bn18 )* , *enpl-1 (RNAi)* N2 , *enpl-1 (RNAi)* *glp-1 ( bn18 )* . To evaluate the percent embryonic lethality, Welch's ANOVA with the Games Howell Post-Hoc Test was performed due to the violation of homogeneous variance assumption in a traditional ANOVA. Since percent of arrested larvae data have normality violation in a traditional ANOVA, a non-parametric Kruskal-Wallis test followed by a Post-Hoc Dunn test was applied. The multiple comparisons were adjusted by the Bonferroni correction.

## Reagents

Worm strains

**Table:** 

**Strain**	**Genotype**	**Available from**
N2	Wild type	*Caenorhabditis* Genetics Center
DG2389	*glp-1 ( bn18 ) *	*Caenorhabditis* Genetics Center

&nbsp;

**&nbsp;**

Plasmids

**Table:** 

**Plasmid**	**Description**
GFP RNAi feeding clone	GFP coding region cloned into L4440 feeding vector in *E. coli* HT115 for *gfp(RNAi)*
*enpl-1* RNAi feeding clone	*enpl-1* ( T05E11.3 ) *C. elegans* genomic clone (1189 bp) from the Ahringer library (Kamath et al., 2003) for *enpl-1 (RNAi) *
